# Simultaneous inhibition of deubiquitinating enzymes (DUBs) and autophagy synergistically kills breast cancer cells

**DOI:** 10.18632/oncotarget.2904

**Published:** 2015-02-27

**Authors:** Rachel Isaksson Vogel, Kathleen Coughlin, Alessandra Scotti, Yoshie Iizuka, Ravi Anchoori, Richard B. S. Roden, Mauro Marastoni, Martina Bazzaro

**Affiliations:** ^1^ Masonic Cancer Center, University of Minnesota, Minneapolis, Minnesota, USA; ^2^ Department of Obstetrics, Gynecology and Women's Heath, University of Minnesota, Minneapolis, Minnesota, USA; ^3^ Department of Pharmaceutical Sciences, University of Ferrara, Ferrara, Italy; ^4^ Department of Pathology, Johns Hopkins University, Baltimore, MD, USA; ^5^ Department of Oncology, Johns Hopkins University, Baltimore, MD, USA; ^6^ Department of Gynecology and Obstetrics, Johns Hopkins University, Baltimore, MD, USA

**Keywords:** Breast Cancer, Triple Negative Breast Cancer, Proteasome Inhibitors, Deubiquitinating Enzymes, Autophagy

## Abstract

Breast cancer is one of the leading causes of cancer death among women in the United States. Patients expressing the estrogen and progesterone receptor (ER and PR) and human epidermal growth factor 2 (HER-2) tumor markers have favorable prognosis and efficacious therapeutic options. In contrast, tumors that are negative for these markers (triple-negative) have a disproportionate share of morbidity and mortality due to lack of a validated molecular target.

Deubiquitinating enzymes (DUBs) are a critical component of ubiquitin-proteasome-system degradation and have been shown to be differentially expressed and activated in a number of cancers, including breast, with their aberrant activity linked to cancer prognosis and clinical outcome. We evaluated the effect of the DUB inhibitors b-AP15 and RA-9 alone and in combination with early- and late-stage lysosomal inhibitors on cell viability in a panel of triple negative breast cancer (TNBC) cell lines.

Our results indicate small-molecule DUB inhibitors have a profound effect on TNBC viability and lead to activation of autophagy as a cellular mechanism to compensate for ubiquitin-proteasome-system stress. Treatment with sub-optimal doses of DUB and lysosome inhibitors synergistically kills TNBC cells. This supports the evaluation of DUB inhibition, in combination with lysosomal inhibition, as a therapeutic approach for the treatment of TNBC.

## INTRODUCTION

Breast cancer is the most frequently diagnosed cancer in women and accounts for over a 40,000 deaths every year in the Unites States alone [1]. Breast cancer can be divided into subtypes based on the expression levels and amplification status of the specific markers estrogen and progesterone receptors (ER and PR) and human epidermal growth factor 2 (HER-2). This distinction is clinically relevant as patients that express ER, PR, and HER-2 have a very favorable prognosis and wider therapeutic options including agents that interfere with hormone production and action or agents that inhibit HER-2 [2–6]. However, tumors that are negative for ER, PR, and HER-2 markers, referred to as triple-negative breast cancer (TNBC), account for about 20% of breast cancer in the Unites States and have a disproportionate share of the morbidity and mortality. This is likely due to its aggressive behavior, increased incidence in younger women, and lack of effective targeted therapies [7–9]. Unfortunately, TNBC is insensitive to some of the most effective breast cancer therapies. Given the lack of validated molecular target and the poor outcome, there is a great need for development of better therapies in patients with TNBC.

The ubiquitin-proteasome-system (UPS) and lysosomal pathway are two major paths for protein degradation within eukaryotic cells, responsible for degradation of short- and long-lived proteins, respectively [10, 11]. Accumulating evidence suggests a crosstalk between these two degradation pathways, with the lysosomal pathway compensating for degradation of short-lived ubiquitinated proteins when USP activity is impaired [12, 13]. It has been previously shown that, independent of the genetic causes leading to malignant transformation, cancer cells exhibit up-regulation of components of protein degradation pathways [14]. This includes up-regulation of deubiquitinating enzymes (DUBs), a critical component of UPS degradation, which are responsible for removal of ubiquitin monomers and chains prior to proteasomal degradation. Members of the DUB family have been shown to be differentially expressed and activated in a number of cancer settings, including breast cancer, with their aberrant activity linked to cancer prognosis and clinical outcome [15–18]. Importantly, we have recently shown that targeting ubiquitin-mediated degradation upstream of proteasomes, whether by targeting DUBs [19–21] or ubiquitin receptors [22] has preclinical efficacy without apparent side effects in preclinical models of ovarian and breast cancer.

Here, we show that treating TNBC cells with the recently identified small-molecule DUB inhibitors b-AP15 and RA-9 has a profound effect on ubiquitin-dependent protein degradation and leads to caspase-3 mediated onset of apoptosis. Mechanistically, b-AP15 and RA-9 treatment leads to autophagy activation as a cellular mechanism to compensate for unsustainable proteotoxic stress in breast cancer cells. Lastly, because misfolded and ubiquitinated proteins are degraded via both proteasomes and lysosomes, simultaneous inhibition of proteasome and lysosomal pathways synergistically induces cell death in TNBC cells. In light of this mechanism, we suggest that evaluation of DUB inhibition alone or in combination with lysosomal inhibition is a novel potential therapeutic approach for treatment of TNBC.

## RESULTS

### Inhibition of proteasome-associated DUBs induces UPS stress in TNBC cells

**Figure 1:**
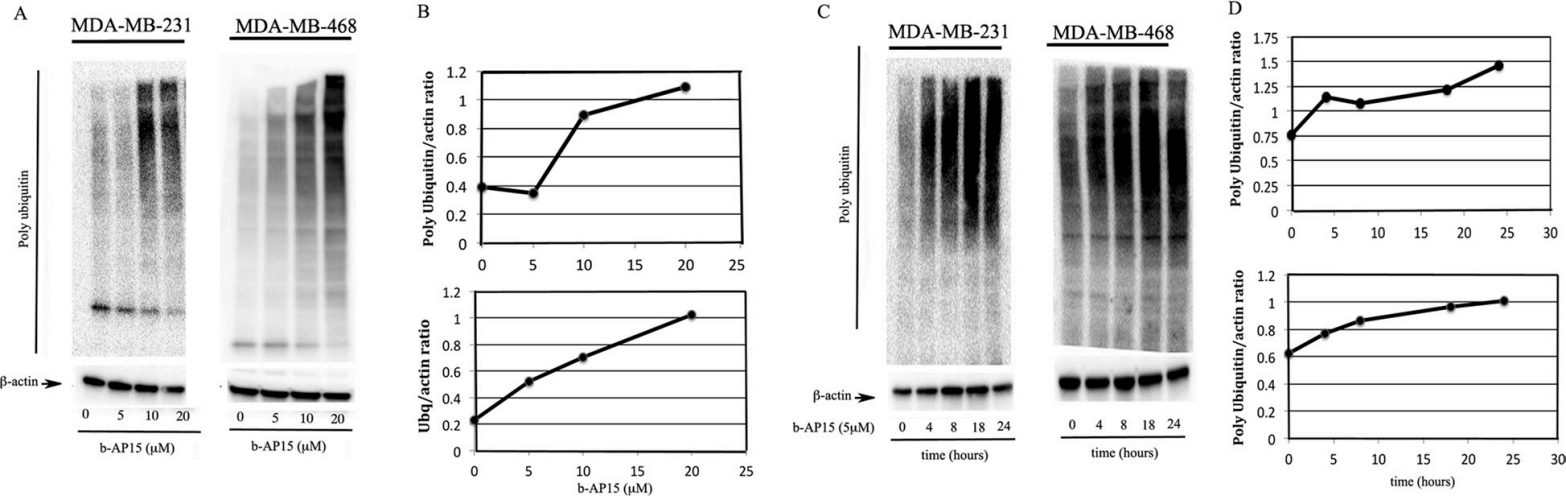
Dose- and time-dependent inhibition of ubiquitin-dependent protein degradation in TNBC cells exposed to b-AP15 **(A)** dose-dependent accumulation of high-molecular weight ubiquitin species in MDA-MB-231 (*left panel*) and MDA-MB-468 (*right panel*) TNBC cell lines exposed to the indicated concentration of b-AP15 for 18 hours. β-actin was used as loading control. **(B)** quantification of the ubiquitin/β-actin ratio for dose-dependent treatment in MDA-MB-231 (*top panel*) and MDA-MB-468 (*bottom panel*) cell lines. **(C)** time-dependent accumulation of high molecular weight ubiquitin species in MDA-MB-231 (*left panel*) and MDA-MB-468 (*right panel*) TNBC cell lines exposed to 5 μM b-AP15 over a period of 24 hours. β-actin was used as loading control. **(D)** quantification of the ubiquitin/β-actin ratio for time-dependent treatment in MDA-MB-231 (*top panel*) and MDA-MB-468 (*bottom panel*) cells.

The effect of the small-molecule DUB inhibitor b-AP15 on ubiquitin-dependent protein degradation was evaluated in the TNBC breast cancer cell lines MDA-MD-231 and MDA-MB-468. Specifically, breast cancer cells were exposed to increasing concentrations of b-AP15 (0–20 μM) over a period of 24 hours; the effect on cellular protein ubiquitination was evaluated by Western blot analysis. As shown in Figure 1A, b-AP15 treatment resulted in a dose-dependent accumulation of poly-ubiquitinated proteins in MDA-MB-231 (*left panel*) and MDA-MB-468 (*right panel*) cell lines starting at 5 μM b-AP15 treatment. Quantification of the changes in high-molecular weight ubiquitin species in each respective cell line, versus control, is given in Figure 1B (*top and bottom panels*). Next, we monitored the rate of poly-ubiquitinated protein accumulation in MDA-MB-231 and MDA-MB-468 breast cancer cell lines exposed to 5 μM b-AP15 over a period of 24 hours. As shown in Figure 1C, immunoblot analysis revealed a rapid, occurring as early as 4 hours from drug exposure, and a time-dependent increase of high molecular weight poly-ubiquitinated species in MDA-MB-231 (*left panel*) and MDA-MB-468 (*right panel*) cells. Quantification of the changes in high-molecular weight ubiquitin species in treated cells versus control is given in Figure 1D (*top and bottom panels*). Taken together, these data suggest that inhibiting proteasome-associated DUBs severely compromises ubiquitin-mediated protein degradation in TNBC cells lines.

### Inhibition of proteasome-associated DUBs is associated with loss of cell viability in TNBC cells via onset of apoptosis

**Figure 2:**
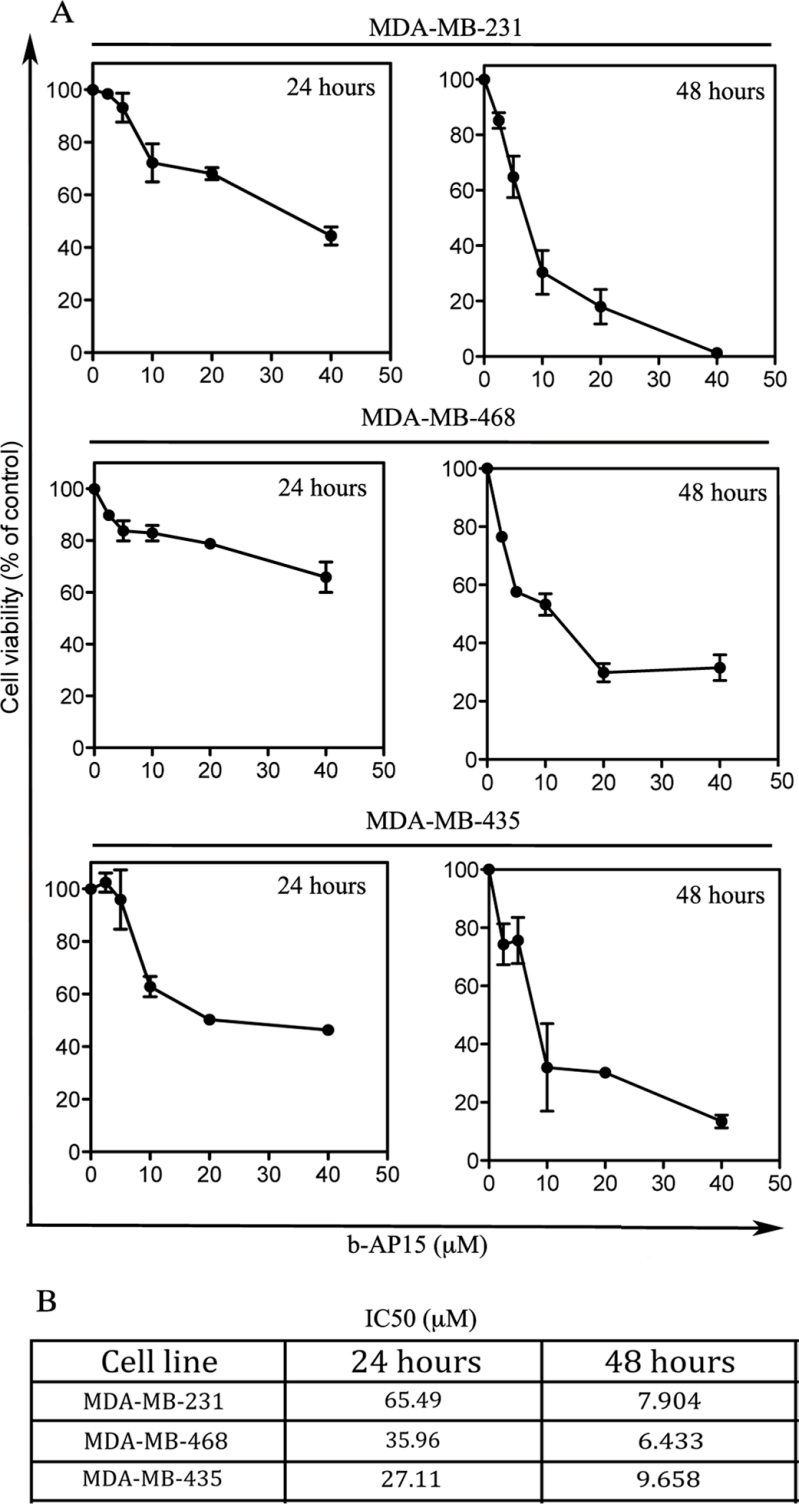
Effect of b-AP15 treatment on TNBC cell viability **(A)** Cell viability of MDA-MB-231, MDA-MB-468 and MDA-MB-435 TNBC cell lines exposed to the indicated concentration of b-AP15 for either 24 or 48 hours. Cell viability was measured by WST-1 assay and is expressed as percent (%) of control. Each experiment was performed in triplicate with means and standard error bars presented; some error bars appear missing due to the small variation between experiments. **(B)** IC50s per each cell line at 24 or 48 hours.

We and others have previously shown that, due to their higher requirement for metabolic activity, cancer cells are highly dependent on proteasome degradation [12, 20]. Therefore, we sought to test whether accumulation of poly-ubiquitinated protein following b-AP15 exposure results in loss of cell viability. To this end, the highly aggressive MDA-MB-231 and MDA-MB-435 and weakly invasive MDA-MB-468 cell lines were exposed to increasing doses of b-AP15 over of 24 and 48 hour period and residual cell viability evaluated by XTT assay. Our results indicate that exposure to increasing concentrations of b-AP15 compromised the cell viability of the breast cancer cell lines tested in a time-dependent fashion (Figure 2A) with the IC50 ranging from ≅10 to ≅6 μM following 48 hours of drug exposure (Figure 2B).

**Figure 3:**
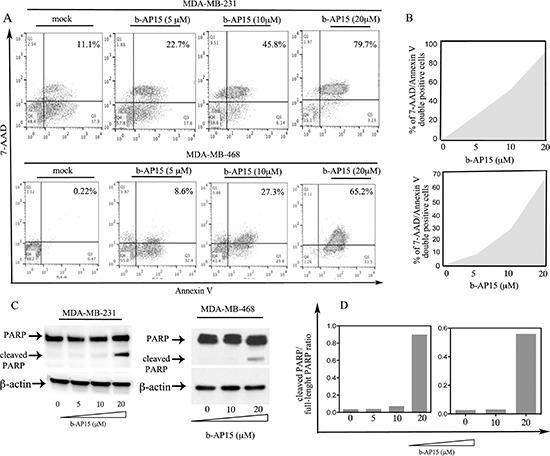
b-AP15 causes onset of apoptosis in breast cancer cells **(A)** MDA-MB-231 (*top panel*) and MDA-MB-468 (*bottom panel*) TNBC cells were either mock treated or treated with b-AP15 at the indicated concentrations for 18 hours. The cells were harvested and stained for Annexin-V and permeability to 7-AAD, a substrate for caspase-3. Percentages of cell double positive are indicated in the *upper right* quadrants. **(B)** quantification of the increase in Annexin-V and 7-AAD double-positive cell populations (shaded area) in MDA-MB-231 (*top panel*) and MDA-MB-468 (*bottom*) cells following b-AP15 treatment. **(C)** cell lysate from MDA-MB-231 (*left panel*) and MDA-MB-468 (*right panel*) TNBC lines exposed to increasing concentrations of b-AP15 over 18 hours via Western blot with an antibody recognizing both the full-length and cleaved forms of PARP. Equivalent protein loading was verified by using an antibody directed against β-actin. **(D)**. Quantification of the cleaved PARP/full-length PARP ratio.

To test whether the reduction in cell viability in TNBC cells following drug exposure is accompanied by the onset of apoptosis, MDA-MB-231 and MDA-MB-468 cell lines were exposed to increasing concentrations of b-AP15 (5–20 μM) over a period of 18 hours. Onset of apoptosis was evaluated via FACS analysis following cell double staining with Annexin V and 7-AAD. As shown in Figure 3A (*top and bottom panel*), exposure to b-AP15 resulted in a dose-dependent increase in the percentage of double positive cells in MDA-MB-231 and MDA-MB-468 cells respectively. Quantification of the increase in Annexin-V and 7-AAD double-positive cell populations following b-AP15 in both cell lines is presented in Figure 3B (*top and bottom panel*).

Finally, we measured the levels of cleaved Poly (ADP-ribose) polymerase (PARP), a substrate of caspase-3, in both cell lines. MDA-MB-231 and MDA-MB-468 cells were exposed to increasing doses of b-AP15 over 18 hours and were analyzed by Western blot using an antibody that recognized both full-length and cleaved PARP. As shown in Figure 3C (*left and right panels*), treatment with b-AP15 resulted in a dose-dependent accumulation of the cleaved formed of PARP with doses of 20 μM in both cell lines. Quantification of the ratio of cleaved PARP to full-length PARP for each dose of b-AP15 in both cell lines is presented in Figure 3D (*left and right panels*). Taken together these data suggest b-AP15 treatment induces loss of cell viability through caspase-3 mediated apoptotic cell death in TNBC cells.

### Inhibition of proteasome-associated DUBs induces onset of autophagy

**Figure 4:**
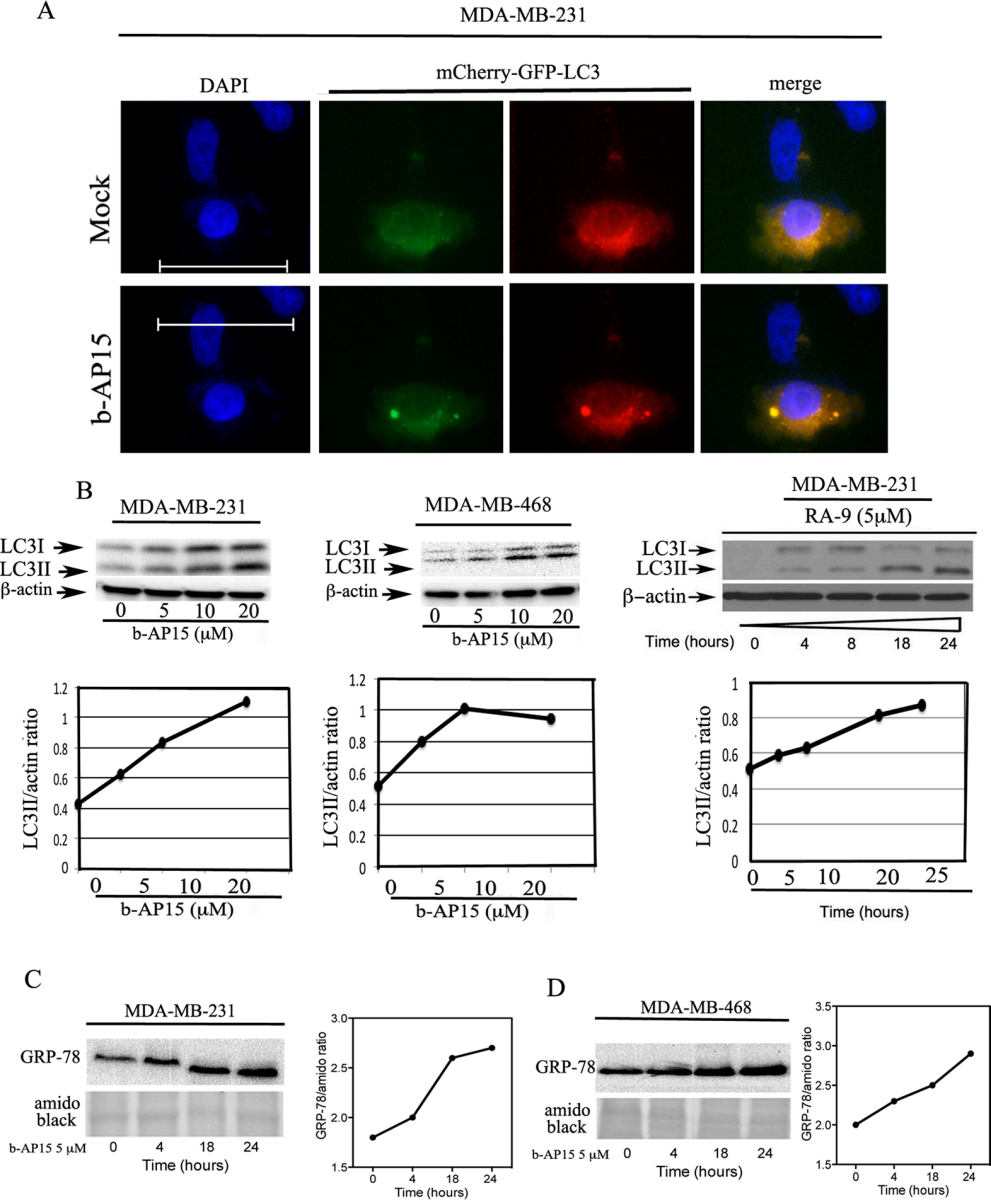
Inhibition of proteasome-associated DUBs induces ER stress responses and onset of autophagy in TNBC cells **(A)** MDA-MB-231 breast cancer cells stably expressing the tandem-tagged mCherry-GFP-LC3 were either mock treated or exposed to 5 μM of b-AP15 over a period of 18 hours and LC3 puncta were visualized by fluorescence microscopy (objective, 60X). **(B)** *left panel*, dose-dependent accumulation of LC3-II isoforms in MDA-MB-231 TNBC cells exposed to the indicated dose of b-AP15 over 18 hours and quantification of the LC3II/β-actin ratio. *Middle panel*, dose-dependent accumulation of LC3-II isoforms in MDA-MB-468 TNBC cells exposed to the indicated dose of b-AP15 over 18 hours and quantification of the LC3II/β-actin ratio. *Right panel*, time-dependent accumulation of LC3-II isoforms in MDA-MB-231 breast cancer cells exposed to 5 μM RA-9 for the indicated time and quantification of the LC3II/β-actin ratio. β-actin was used as loading control. **(C)** *Left panel*, MDA-MB-231 breast cancer cells exposed to 5 μM of b-AP15 over a period of 24 h following Western blot analysis with specific antibody against the ER stress-associated proteins GRP-78, amido black was used as loading control. *Right panel*, quantification of the ER stress-associated proteins/amido black ratio. **(D)** *Left panel*, MDA-MB-468 breast cancer cells exposed to 5 μM of b-AP15 over a period of 24 h following Western blot analysis with specific antibody against the ER stress-associated proteins GRP-78, amido black was used as loading control. *Right panel,* quantification of the ER stress-associated proteins/amido black ratio.

We have previously shown that inhibition of proteasome-associated DUBs via small-molecule inhibitors induce endoplasmic reticulum (ER) stress response as a mechanism to compensate for unsustainable proteotoxic stress [23]. Here, we evaluated whether inhibition of proteasome-associated DUBs with the specific inhibitors b-AP15 or RA-9 would trigger autophagy as a cytoprotective response in TNBC cells. MDA-MB-231 cells were transiently transfected with the mCherry-GFP-LC3 reporter and exposed to mock (control) or 5 μM b-AP15 treatment for 18 hours. Onset of autophagy was visualized by immunofluorescence microscopy. As shown in Figure 4A, TNBC cells treated with b-AP15 displayed a punctate LC3 localization characteristic of autophagosome formation. Autophagy activation following inhibition of proteasome-associated DUBs was further confirmed by measuring protein expression levels of the LC3-I and LC3-II isoforms by Western blot analysis. During autophagy, the cytosolic form of LC3 (LC3-I) is conjugated to phosphatidylethanolamine to form LC3-phosphatidylethanolamine conjugate (LC3-II), which is recruited to autophagosomal membranes. This causes accumulation of the LC3-II isoform over the LC3-I isoform [24]. Here, MDA-MB-231 and MDA-MB-468 TNBC cells were exposed to increasing doses of b-AP15 over 18 hours and analyzed by Western blot with an anti-LC3 antibody to assess levels of both LC3-I and its lipidated, autophagic vescicle-associated LC3-II isoform [25, 26]. Consistent with our initial hypothesis of autophagy activation, b-AP15 treatment caused dose-dependent accumulation of LC3-II isoforms in each cell line (Figure 4B, *left and middle panels*) and RA-9 treatment caused time-dependent accumulation of LC3-II isoforms in MDA-MB-231 (Figure 4B, *right panel*). We have recently shown that inhibition of ubiquitin-dependent protein degradation upstream of proteasome causes activation of ER stress responses in ovarian cancer cells [27]. To test whether a similar event occurs in TNBC, MDA-MB-231 and MDA-MB-468 cells were exposed to 5 μM b-AP15 over a period of 24 h and the cell lysates subjected to Western blot analysis for the ER-stress marker GRP-78. We found that b-AP15 exposure caused a time-dependent increase in the steady levels of GRP-78 in both MDA-MB-231 (Figure 4C) and MDA-MB-468 cells (Figure 4D). Quantification of the changes in the steady state levels of GRP-78 in MDA-MB-231 and MDA-MB-468 cells are provided in the right panels of Figures 4C and 4D, respectively.

**Figure 5:**
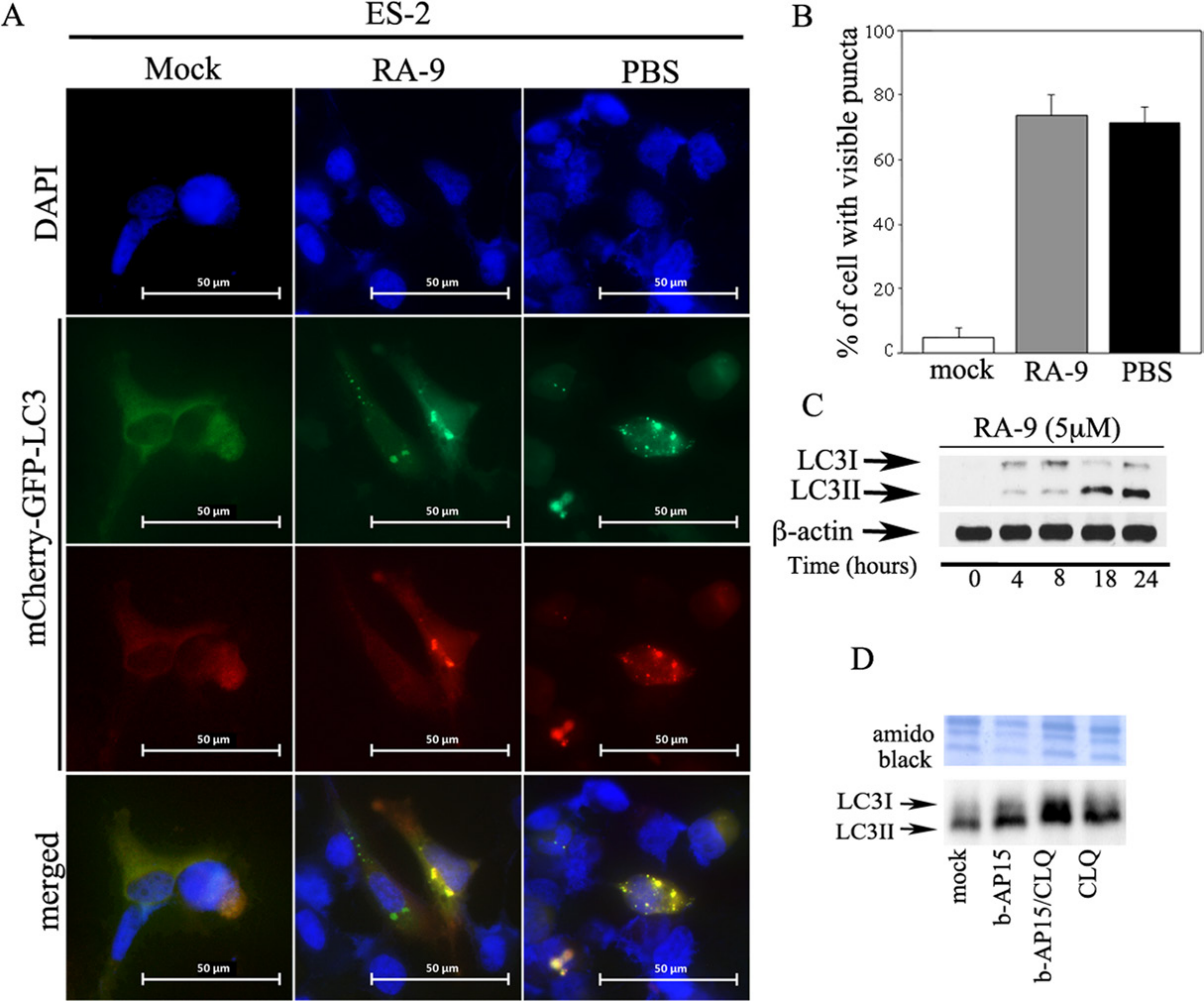
Inhibition of proteasome-associated DUBs induces autophagic flux in cancer cells **(A)** ES-2 ovarian cancer cells stably expressing the tandem-tagged mCherry-GFP-LC3 were either mock treated or exposed to 5 μM of RA-9 over a period of 18 hours and LC3 puncta were visualized by fluorescence microscopy. PBS was used as positive control autophagy inducer (objective, 60X). **(B)** quantification of the number of cells with visible puncta in treated versus controls. **(C)** dose-dependent accumulation of LC3-II isoforms in ES-2 cells exposed to the indicated dose of RA-9 over 24 hours and quantification of the LC3II/β-actin ratio. β-actin was used as loading control. **(D)** autophagy flux measured in ES-2 ovarian cancer cells either mock treated or treated with 5 μM b-AP15 alone, combination of 5 μM b-AP15 and 50 μM of the autophagy inhibitor Chloroquine or 50 μM of the autophagy inhibitor Chloroquine alone over a period of 18 hours.

Next, we wanted to test whether tested whether activation of autophagy following DUB inhibition is a unique event in breast cancer cells or a common feature in other cancer types. To this end, ES-2 ovarian cancer cells were exposed to mock, 5 μM RA-9 treatment for 18 h, or, as a positive control, autophagy was induced by amino acid starvation in presence of PBS for a period of 3 h. As shown in Figure 5A, both amino acid starved and RA-9 treated ovarian cancer cells displayed punctate LC3 localization characteristic of autophagosome formation. Figure 5B show the percent of cells that contained visible puncta per each condition. Consistent with the observation of increased LC3-II levels in TNBC cells following DUB inhibition, RA-9 treatment also resulted in increased levels of the LC3-II lipidated form in the ovarian cancer cell. (Figure 5C). However, because LC3-II degradation occurs via autophagy, stabilization of its lipidated isoform could be the result of autophagy inhibition rather than its activation. To rule out this possibility, we measured the autophagic flux cancer cells treated with either b-AP15 and the autophagy inhibitor Chloroquine alone or in combination. As shown in Figure 5D, blocking the last step of autophagic flux with Chloroquine prevented the lysosomal degradation of LC3-II in autophagosomes, resulting in further LC3-II isoform accumulation in the ovarian cancer cells. Taken together this strongly suggests that inhibition of protein-associated DUBs causes onset autophagy flux as an alternative pathway to proteasomal degradation [28, 29].

### Synergistic effect of DUB and autophagy inhibitors in inducing apoptosis in TNBC cells

**Figure 6:**
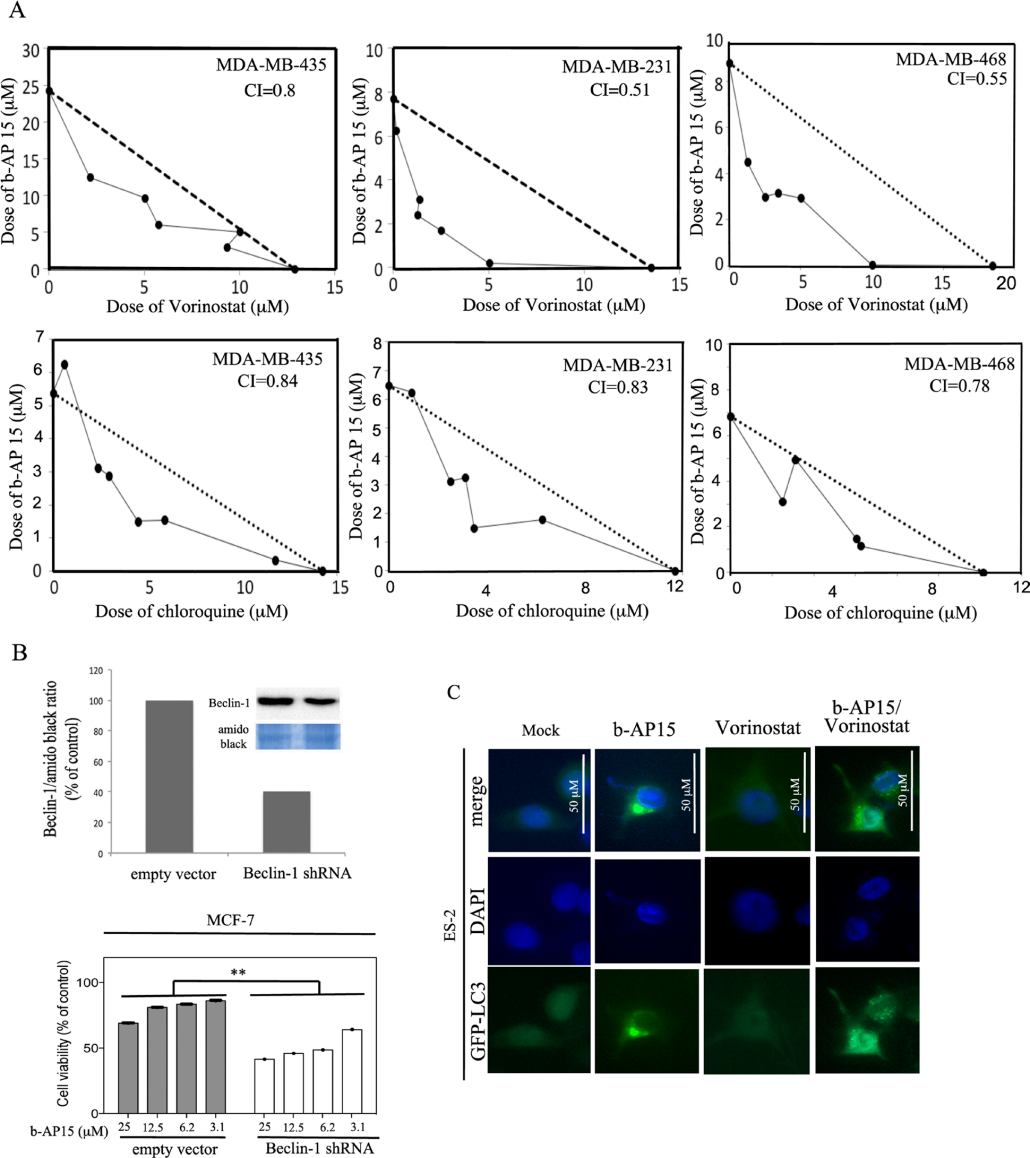
DUB and autophagy inhibitors synergistically kill TNBC cells **(A)** Cultures of MDA-MB-435 (*left panel*), MDA-MB-231 (*middle panel*), MDA-MB-468 (*right panel*) were treated with checkerboard dilutions series of b-AP15 and Vorinostat (*top panel*) and Chloroquine (*bottom panel*). Cell viability was measured by XTT assay and calculated as percent of control untreated cultures. Synergy was shown by plotting the interaction between drugs in isobolograms. The dotted diagonal line corresponds to an additive effect; points below the diagonal indicate synergy. The combination index (CI) is provided; CI < 1 indicates synergism, CI = 1 indicates additivity, and CI > 1 indicates antagonism. **(B)** *Top panel*, quantification of Beclin-1 protein expression levels in MCF-7 breast cancer cells stably expressing empty vector or Beclin-1 shRNA as measured by Western blot analysis (*insert*). *Bottom panel*, dose-dependent inhibition of the cell viability MCF-7 breast cancer cells expressing empty vector or Beclin-1 shRNA in presence of the b-AP15 DUB inhibitor at the indicated concentrations. Cell viability was measured after a 48-h incubation by WST-1 assay and the percentage of viable cells is presented relative to mock-treated controls. **, *P* < 0.01. **(C)** GFP-LC3 expressing ES-2 ovarian cancer cells were either mock treated or incubated with 5 μM b-AP15, 20 μM of Vorinostat or the combination of both for 18 h before fixation and immuno-fluorescent staining of DNA (blue) and LC3 (green).

Recent data suggest that, in cancer, proteasome- and lysosome-assisted protein degradation are functionally coupled. [20, 28] Our data indicate that following proteasome-associated DUB inhibition, TNBC cells activate autophagy as a protective mechanism to decrease levels of proteotoxic stress. Therefore, we hypothesized that concomitant inhibition of DUBs and autophagy would synergistically trigger cell death. Specifically, we tested the effect of exposing TNBC cell lines, including MDA-MB-435, MDA-MB-231 and MDA-MB-468, to the combination of the proteasome-associated inhibitor b-AP15 and Vorinostat or Chloriquine over a period of 48 hours. As shown in Figure 6A, analyses of cell death indicated synergistic activity for the b-AP15 and Vorinostat combination in all of cell lines tested, with the CI of 0.80, 0.51 and 0.55 for MDA-MB-435, MDA-MB-231 and MDA-MB-468, respectively. Likewise, the combination of b-AP15 and the clinically approved lysosome inhibitor Chloroquine shows synergistic cell killing with CIs of 0.84, 0.83 and 0.78 for MDA-MB-435, MDA-MB-231 and MDA-MB-468, respectively, though to a lesser extent. These data indicate that rather than a simple additive killing, the combination of a DUB and lysosome inhibitor is highly to moderately synergistic.

To further confirm that inhibition of autophagy is responsible for the increased cell killing observed following DUB inhibition and Vorinostat and Chloroquine treatment, we took advantage of knock-down technology to selectively knock-down Beclin-1, which is known to regulate autophagy by promoting the fusion of the autophagosomes to the lysosomes [29]. Specifically Beclin-1 was knocked down in MCF-7 breast cancer cells and the effect on protein levels was evaluated via Western blot analysis. As shown in Figure 6B (*top panel*) Beclin-1 expression levels were reduced by 60% in Beclin-1 shRNA cells as compared to control. Next, Beclin-1 and control knockdown cells were exposed to escalating concentrations of the DUB inhibitor b-AP15 over a period of 48 hours and residual cell viability measured via WST-1 assay as we previously described [27]. As shown in Figure 6B (*bottom panel*) Beclin-1 knockdown MCF-7 breast cancer cells show a statistically significant higher degree of sensitivity to b-AP15 as compared to the parental cells. Importantly, the MCF-7 breast cancer cell line expresses both the estrogen and the progesterone receptor, thus suggesting that concomitant inhibition of DUBs and autophagy could be used as a therapeutic option for patients whose tumors are hormone sensitive.

We have previously shown that activation of autophagy is a cytoprotective event to limit proteasome inhibitor-induced cell death [13] and that inhibition of proteasome-associated protein degradation via proteasome inhibitors is associated with the formation of aggresomes. Therefore we performed immunofluorescence analysis to investigate the fate of poly-ubiquitinated protein following DUB inhibition. Specifically, the GFP-LC3 expressing ES-2 ovarian cancer cell line was treated with b-AP15 (5 μM), Vorinostat (20 μM) or combination of both for 18 hours and the sub-cellular localization of LC3, was visualized by immunofluorescence. As shown on Figure 6C, b-AP15 treatment led to the visualization of LC3 localization characteristic of autophagosome formation while Vorinostat treatment did not cause significant LC3 accumulation. Simultaneous treatment with b-AP15 and Vorinostat, on the other hand, caused the appearance of poly-ubiquitinated proteins at multiple punctate sites throughout the cytoplasm. This is consistent with a cytoprotective role of autophagy following DUB inhibitor treatment.

## DISCUSSION

Independent of the genetic components that predispose individuals to breast cancer, malignant transformation is accompanied by progressive cellular adaptation to cope with increasing levels of metabolic stress inherent to the cancer phenotype [12–14, 30, 31] and targeting of these compensatory mechanisms results in selective killing of cancer cells.

DUBs are a large family of proteases responsible for removing ubiquitin (monomers and chains) from the substrates prior to their degradation by proteasomes. This is needed for proper protein homeostasis as de-ubiquitination is essential for degradation of proteins by proteasomes. DUBs represent attractive and novel “drug-able” targets for cancer as they are substrate specific and directly responsible for controlling the steady levels of proteins crucial for maintaining the malignant phenotype. Recent evidence suggests a link between activation status of certain DUB family members and cancer progression and chemoresistance in a number of cancer settings, including breast cancer [16–18], thus supporting the concept of DUBs playing a crucial role in cancer development and its outcome. Studies have also suggested that pharmacological inhibition of DUBs decreases tumor burden and increases overall survival in xenograft models of human cancers [15, 22, 32–34], supporting the feasibility of targeting DUBs for cancer treatment

To assess the feasibility of inhibiting DUBs for TNBC treatment we initially evaluated the effect of the recently identified small-molecule DUB inhibitors RA-9 and b-AP15 on ubiquitin-dependent protein degradation and cell viability in a panel of TNBC cell lines. Our data show that protracted UPS stress following exposure to suboptimal concentration of DUB inhibitors cause dose- and time dependent proteotoxic stress in TNBC cell lines which is consistent with a decrease in cell viability at pharmacologically achievable concentrations.

A number of recent studies suggest that DUBs are responsible for deubiquitinating of a number of cell cycle regulators and consequent regulation of their steady state levels [35]. For instance, DUBs have been proposed to play a critical role in regulating NF-κB pathway [36], TGFβ signaling [37] as well as p53 and MDM2 [38] levels and activity suggesting that DUB dysregulation is a frequent event in cancer.

Our present study shows that treatment of TBNC cells with the DUBs inhibitor b-AP15 causes dose-dependent caspase-3 activation and apoptosis both of which occurs well after onset of proteotoxic stress, suggesting that apoptosis mediated cells death is a consequence of unsustainable levels of UPS stress in breast cancer cells following DUBs inhibition.

The proteasome- and lysosome-mediated protein degradation are the two mechanisms for protein degradation within eukaryotic cells. Under physiological circumstances these two evolutionarily conserved mechanisms for protein degradation work independently, the first being responsible for the degradation of over 90% of short-lived proteins the latter being responsible for degradation of long-lived proteins. A cross-talk between proteasome- and lysosome-mediated protein degradation has been suggested in cancer, signifying that proteasome- and lysosome-assisted protein degradation are functionally coupled.

In support of this hypothesis, our results indicate treatment of TNBC cells with sub-optimal concentrations of the DUB inhibitors b-AP15 and RA-9 causes activation of autophagy as a mechanism for cells to escape to unsustainable levels of UPS stress. Importantly activation of autophagy occurs well before onset of apoptosis, suggesting that activation of autophagy is a protective cellular mechanism to decrease the levels of proteotoxic stress rather than cause cellular death.

Lastly, we wanted to explore whether pharmacological inhibition of proteasome-associated DUBs have the potential as part of combination treatment for TNBC patients. Our results indicate that pharmacological inhibition of DUB function via the small-molecule b-AP15 and of autophagy via Vorinostat or Chloroquine induces synergistic cell killing in a panel of TBNC cells with CIs ranging from 0.51–0.84, providing justification for further exploration of this combinatorial treatment as a therapeutic agents for TNBC.

## METHODS

### Reagents and plasmids

The DUB inhibitors b-AP15 and RA-9 were synthetized as previously described [19, 32]. The 2, 3-bis[2-methoxy-4-nitro-5-sulfophenyl]-2H-tetrazolium-5-carboxanilide inner salt (WST-1) was purchased from Cayman Chemicals. The autophagy inhibitors Vorinostat and Chloroquine were purchased from Sigma. The mCherry-GFP-LC3 plasmid was kindly provided by Dr. Michael K Lee (University of Minnesota, MN).

### Cell lines and transfection

The cell lines MDA-MB-435, MDA-MB-231 and MDA-MB-468 were a generous gift from Drs. Deepali Sachdev and Vitaly Polunovsky (University of Minnesota, MN) and were cultured in DMEM supplemented with 10% fetal bovine serum, 100 IU/mL penicillin, and 100 μg/mL streptomycin at 5% CO_2_. The MCF-7 breast cancer cell line stably expressing empty vector or Beclin-1 shRNA were a generous gift from Dr. Ameeta Kelekar and were cultured as previously described [29]. The origin of the MDA MB 435 cell line is controversial. [39, 40] Here we have used it as a model of a highly metastatic cell line. The ovarian cancer cell line ES-2 was obtained from American Type Cell Culture and cultured as described above. For immunofluorescence experiments, subconfluent cultures of MDA-MB-231 cells were transiently transfected with the mCherry-GFP-LC3 plasmid using Lipofectamine 2000 reagent (Life Technologies) according to the manufacturer's instructions.

### Cell viability assay

Cell viability was determined by 2,3-bis[2-methoxy-4-nitro-5-sulfophenyl]-2H-tetrazolium-5-carboxanilide inner salt (WST-1, Cayman Chemicals) assay as previously described [19, 41]. Briefly, cells were seeded at the concentration of 1,000 per well in 100 μL medium in 96-well plate and treated with the indicated concentrations of drugs. At the indicated time points, cells were incubated according to the manufacturer's protocol with the WST-1 labeling mixture for 2 hours. Formazan dye was quantified using a spectrophotometric plate reader to measure the absorbance at 450nm (ELISA reader 190; Molecular Devices).

### Antibodies and western blot analysis

Total cellular protein (10–20 μg) from each sample was separated by SDS-PAGE, transferred to PVDF membranes and subjected to Western blot analysis. Antibodies for Western blot analysis were obtained by the following commercial sources: anti-ubiquitin (Santa Cruz Biotechnology), anti-PARP (BD Pharmingen), anti-LC3 (Cell Signaling) anti–β-actin (Sigma), anti-Beclin-1 (Abcam), anti-GRP-78 (Cell signaling). Peroxidase-linked anti-mouse Immunoglobulin G and peroxidase-linked anti-rabbit Immunoglobulin G were from Amersham.

### Immuno-fluorescence analyses

For analysis of LC3 puncta, mCherry-GFP-LC3 expressing MDA-MB-231 or ES-2 were grown as described above in Lab-Tek II chambered cover glass (Nalge Nunc International) and treated with the indicated concentrations of drugs. Following fixation and permeabilization with cold methanol, DNA was visualized by 4,6-diamidino-2-phenylindole (DAPI) staining and mounted samples were viewed under a Nikon Eclipse TE 2000E inverted microscope. Images were captured with Spot 3.5.8 acquisition software (Diagnostic Instruments).

### Determination of apoptotic cells by flow cytometry

Annexin-V/7-AAD and active caspase-3 staining methods were used to determine induction of apoptosis. Annexin-V/7-AA D staining was conducted using Annexin V-PE Apoptosis Detection Kit I (BD Pharmingen) according to the manufacturer's protocol and as we have previously described [12]. Active caspase-3 staining was done using phycoerythrin-conjugated rabbit anti-active caspase-3 monoclonal antibody (BD PharMingen) according to the manufacturer's protocol and as previously described [12]. Data analysis was performed using CellQuest software (Becton Dickinson Immunocytometry System).

### Statistical analysis

The combination index (CI) of b-AP15 and Vorinostat or Chloriquine was calculated by the method of Chou and Talalay [42] and further regression analyses were performed to stabilize estimates. CI < 1 indicates synergism, CI = 1 indicates additivity, and CI > 1 indicates antagonism.
